# Evaluation of Carotid Artery Intima-Media Thickness as a Cardiovascular Risk Factor in Patients With Polycystic Ovary Syndrome

**DOI:** 10.7759/cureus.13025

**Published:** 2021-01-31

**Authors:** Pinar Yalcin Bahat, Alper Özel, Alper Demirci

**Affiliations:** 1 Obstetrics and Gynecology, Kanuni Sultan Süleyman Training and Research Hospital, Istanbul, TUR; 2 Radiology, Şişli Hamidiye Etfal Training and Research Hospital, Istanbul, TUR

**Keywords:** carotid artery intima-media thickness, polycystic ovary syndrome, insulin resistance

## Abstract

Introduction

The purpose of this study is to assess cardiovascular risk factors in patients diagnosed with polycystic ovary syndrome (PCOS) by comparing dyslipidemia, insulin sensitivity, hyperhomocysteinemia, carotid artery intima-media thickness (CIMT) between women diagnosed with PCOS and healthy subjects.

Materials and Methods

Hundred women diagnosed with PCOS aged between 18 and 35 years and who applied to tertiary center gynecology clinic were included in the study. Hundred women who applied for menstrual irregularity to the same outpatient clinic with no systemic diseases, who were not under medical treatment for any condition, were included in the control group. Physical examination, hormone profile tests on the second day of the patients’ menstrual cycle, pelvic, and neck ultrasonography to evaluate CIMT were performed for all patients. CIMT values were compared with biochemical, hormonal, and anthropometric values. Sensitivity, specificity, Pearson correlation coefficient, mean, and standard deviation were calculated.

Results

In the PCOS group, there was no statistically significant difference in homeostatic model assessment for insulin resistance (HOMA-IR) (<2.5 and >2.5) among all parameters. When we evaluate body mass index (BMI) (<30 and >30) for this PCOS group patients, a very highly significant difference (p < 0.001) between waist-hip ratio and hemoglobin A1c (HbA1c) was established statistically, and there was a significant difference (p < 0,05) between waist-hip ratio and luteinizing hormone (LH).

After this statistical analysis, dehydroepiandrostenedione sulfate (DHEAS), free androgen index (FAI), LH, low-density lipoprotein (LDL), Ferriman-Gallwey score (FGS), homocysteine, mean CIMT, and waist to hip ratio were significantly different in the two groups (p < 0.01). The difference between HbA1c and cholesterol high-density lipoprotein (HDL) was significant (p < 0.05).

Conclusion

As a result, in the PCOS group, when compared to the healthy subjects, dyslipidemia, HbA1c, waist to hip ratio, and CIMT were significantly different. It might be suitable to perform an ultrasound for CIMT in patients with higher Ferriman-Gallwey (FG) score.

## Introduction

Polycystic ovary syndrome (PCOS) is a condition associated with chronic anovulation, insulin resistance (IR), and androgen excess. It is defined as a multiple metabolic syndrome, which is associated with obesity, dyslipidemia, endothelial dysfunction, decreased vessel compliance, and early carotid artery atherosclerosis [1]. PCOS is seen in 6%-10% of women of reproductive age [2]. Up to 70% of women with PCOS have IR, which is considered the cause of hyperinsulinemia and hyperandrogenism (HA) [3].

Ultrasonographic evaluation of carotid artery intima-media thickness (CIMT) is now a well-established and accepted marker of atherosclerosis and is associated with increased cardiovascular risk [4]. It was calculated that each 0.10 mm increase in CIMT, increased myocardial infarction (MI) risk by 15%, and stroke risk by 18% [5]. In other studies, a 0.16 mm increase in CIMT was reported to increase the risk of MI, stroke, and death by 24%, and 19% after correction for age and sex [6]. Therefore, CIMT can be used to identify women with high cardiovascular risk. In patients with increased CIMT diet, lifestyle changes or drug therapy (metformin, cholesterol-lowering agents, etc.) can be administered for preventative measures.

In this study, dyslipidemia, IR, hyperhomocysteinemia, and CIMT measurements as cardiovascular risk factors in patients with PCOS were compared with healthy women in the same age group to investigate whether there was a difference in regarding these parameters in patients diagnosed with PCOS.

## Materials and methods

Patients who were diagnosed with PCOS between 18 and 35 years of age and who visited our tertiary gynecology clinic between February 2015 and August 2015 were included in the study. The control group was chosen randomly from patients who visited the same clinic during the same period of time, who had similar body mass index (BMI), and who were within the same age group.

Patients with the androgen-releasing tumors, diabetes mellitus, hypertension, renal failure, acute or chronic infectious diseases, late-diagnosed congenital adrenal hyperplasia, Cushing’s syndrome, thyroid dysfunction, goiter, hyperprolactinemia, systemic diseases, and those taking oral contraceptives, anti-lipidemic agents, hypertension drugs, and insulin-sensitizing drugs were excluded from the study. Folic acid, vitamin B12, and B6 users in the last six months were not included in the study because these supplements have a known effect on serum homocysteine levels.

PCOS was diagnosed according to the 2003 Rotterdam criteria [7]. Follicle-stimulating hormone (FSH), luteinizing hormone (LH), estradiol (E2), total and free testosterone levels, androstenedione, dehydroepiandrostenedione sulfate (DHEAS), sex hormone-binding globulin (SHBG), prolactin, thyroid-stimulating hormone (TSH), free T3, and free T4 levels, hemoglobin A1c (HbA1c), blood lipid profile, liver function tests, renal function tests, high-sensitivity C-reactive protein (hs-CRP), and homocysteine levels were evaluated for each patient in both groups. Free androgen index (FAI) was calculated; 75 grams of oral glucose loading test was applied (0-, 60-, and 120-minute values), and also fasting insulin values were determined.

Hormone levels were measured with electrochemiluminescent immunoassay (ECLIA), Elecsys, and Cobas e (Roche Hitachi [Roche Diagnostics, Basel, Switzerland]) immunological test analyzers. Plasma glucose levels, HDL cholesterol, triglyceride, LDL cholesterol, and HbA1c were measured in vivo using Roche Hitachi Cobas c systems (Cobas c 311, Cobas c 501/502).

The number of antral follicles (2-9 mm) in both ovaries and both ovary volumes were recorded using transvaginal ultrasonography within the early follicular period for each patient (2-4 days of menstrual period). BMI, systolic, and diastolic blood pressures of all patients were recorded. In addition, homeostatic model assessment for insulin resistance (HOMA-IR) and Ferriman-Gallwey (FG) scoring for hirsutism assessment were performed to evaluate insulin resistance.

Carotid artery ultrasonography was performed by the same radiologist in both groups, and the values were recorded. Bilateral carotid ultrasound was performed with a high-resolution ultrasound system with a 7.5-MHz linear array scan (Toshiba SSH-140A [Toshiba Medical Systems, Tokyo, Japan]). Longitudinal images of both common carotid arteries were obtained from the distal portion. Two bright echogenic lines in the arterial wall were defined as intima and media lines. CIMT was measured as the distance between these echogenic lines. Only posterior carotid wall measurements were recorded. Measurements were made 1-2 cm proximal to the bifurcation. Three measurements were taken from each artery, and mean values were calculated.

Power analysis was performed using Statistical Power Analysis (courtesy of Samuel Borenstein). Statistical analysis was performed using SPSS 23.0 (SPSS Inc., Chicago, IL, USA). One-way Kolmogorov-Smirnov test was used to evaluate the distribution of data. Since the normal distribution was determined between the two groups, student’s t-test was used. All results were expressed as mean ± standard deviation. The relationship between variables was evaluated with Pearson’s correlation test. Two-way analysis of variance (ANOVA) was used to evaluate the effects of obesity in the control and the study group. P ˂ 0.05 value was accepted as statistically significant.

## Results

A total of 200 patients, 100 subjects in the PCOS (study) group and 100 subjects in the control group, were included in the study. The demographic characteristics of the two groups are shown in Table 1. When the demographic data of the study and control groups were compared, the two groups were similar in terms of their age, BMI, fasting blood glucose, insulin levels, and diastolic blood pressure values.

**Table 1:** Demographic distribution and statistical differences of PCOS and control groups PCOS, polycystic ovary syndrome; BMI, body mass index; HDL, high-density lipoprotein; LDL, low-density lipoprotein; HbA1c, hemoglobin A1c; FAI, free androgen index; HOMA-IR, homeostatic model assessment for insulin resistance; CIMT, carotid artery intima-media thickness.

	PCOS (n = 100)	Control (n = 100)	p value
Age	24.84 ± 5.40	25.19 ± 4.53	0.620
BMI	25.88 ± 3.91	25.07 ± 3.89	0.142
Waist-hip ratio	0.67 ± 0.04	0.70 ± 0.05	<0.001
HDL	52.53 ± 15.30	60.42 ± 17.25	0.001
LDL	95.20 ± 25.62	81.66 ± 18.63	<0.001
Triglyceride	128.37 ± 37.93	125.89 ± 114.32	0.837
Cholesterol	200.05 ± 36.28	187.74 ± 36.07	0.017
Homocysteine	10.04 ± 2.16	8.17 ± 2.00	<0.001
Systolic blood pressure	106.14 ± 9.17	109.05 ± 9.66	0.028
Diastolic blood pressure	66.45 ± 5.65	66.80 ± 5.05	0.645
HbA1c	5.55 ± 0.34	5.42 ± 0.32	0.010
Fasting blood glucose	79.97 ± 8.19	78.70 ± 7.27	0.247
FAI	3.50 ± 3.37	1.71 ± 1.47	<0.001
Ferriman–Gallwey score	17.60 ± 5.59	4.03 ± 1.01	<0.001
Insulin	16.14 ± 12.11	14.25 ± 11.19	0.251
HOMA-IR	3.22 ± 2.53	2.76 ± 2.19	0.176
Right CIMT	0.51 ± 0.12	0,.3 ± 0.07	<0.001
Left CIMT	0.51 ± 0.12	0.43 ± 0.07	<0.001
Mean CIMT	0.51 ± 0.12	0.43 ± 0.07	<0.001

The mean CIMT was 0.51 mm in the study group and 0.43 mm in the control group. A statistically significant difference was found between the two groups (p < 0.001). In addition, the levels of homocysteine, LDL, cholesterol, FG score, and FAI were significantly elevated in the PCOS group. 

When the correlation analysis between CIMT and parameters of cardiovascular risk factors were evaluated as shown in Table 2, a significant correlation was observed only between CIMT and FG score (p < 0.001, r = 0.489). For the rest of the parameters, the correlation was insignificant.

**Table 2:** Correlation table between CIMT and other parameters * indicates the significant correlation observed only between CIMT and FGS. BMI, body mass index; DHEAS, dehydroepiandrostenedione sulfate; SHBG, sex hormone-binding globulin; HDL, high-density lipoprotein; LDL, low-density lipoprotein; HOMA-IR, homeostatic model assessment for insulin resistance; HbA1c, hemoglobin A1c; FGS, Ferriman-Gallwey score; FAI, free androgen index; CIMT, carotid artery intima-media thickness.

	Mean CIMT (p value)
Age	0.097
BMI	0.036
Fasting blood glucose	0.004
DHEAS	0.064
SHBG	0.163
Total testosterone	0.193
HDL	0.053
LDL	0.069
Total cholesterol	0.093
Triglyceride	0.096
HOMA-IR	0.061
HbA1c	0.057
Homocysteine	0.040
FGS	0.489*
Systolic blood pressure	0.021
Diastolic blood pressure	0.131
Waist-hip ratio	0.060
FAI	0.012

## Discussion

PCOS, IR, dyslipidemia, and increase in androgen levels are factors that increase the risk for cardiovascular disease [8]. Risk factors associated with PCOS emerge in early adolescence and play an important role in the development of subclinical atherosclerosis, which can cause cardiovascular diseases [9]. Epidemiological and clinical studies have shown that CIMT is associated with these cardiovascular diseases and is seen as a marker of atherosclerosis [10]. In this study, CIMT did not correlate with other cardiovascular risk factors.

Metabolic abnormalities such as obesity, IR, and hyperandrogenism (HA) are among the risk factors that cause an increase in CIMT [11]. When we compared CIMT measurements between the PCOS group and the control group, we found that CIMT values were higher in the PCOS group. Talbott et al. [12] evaluated CIMT levels in PCOS patients according to different age groups and showed that age had no significant effect on CIMT. In our study, the patients’ age group was 18-35 years, and regardless of age, a significant difference was observed in CIMT values between patients and the control group.

Orio et al. [13] showed a strong positive correlation between CIMT and FAI. This suggests that HA may lead to atherosclerosis in patients with PCOS. On the contrary, in the same study [13], a negative correlation was found between serum DHEAS and androstenedione levels and CIMT. Vryonidou et al. [14] showed that high DHEAS concentrations in PCOS patients were associated with thin intima-media thickness and argued that high serum DHEAS levels had a cardioprotective effect. In contrast, another study [15] showed no protective effect in terms of cardiovascular risk when 900 postmenopausal women with high serum DHEAS levels were examined. In another study [16], 182 postmenopausal women were examined, and no correlation was found between serum DHEAS level and CIMT. In our study, we found a significant positive correlation between CIMT and FG score. There was no significant relationship between serum DHEAS levels. Given that FG score reflects HA, its effect on the cardiovascular system can be meaningful.

IR also plays a critical role in the pathogenesis of PCOS. Especially in obese patients with PCOS, there is a significant increase in IR and type 2 diabetes. In a Hong Kong study [17], it was reported that IR, abdominal obesity, and oxidative damage are vital in the pathogenesis of PCOS. Chen et al. [18] showed that in women with PCOS, there was an excessive increase in glucose transporter type-4 (GLUT-4) production over time due to increased IR and that this has an important place in metabolic regulation disorder. They compared the young obese group with PCOS to a healthy group and showed that the production of GLUT-4 increased [2] in the PCOS group. Similarly, Carvalho et al. [19] showed that GLUT-4 production decreased in the group without IR. However, it was observed that PCOS and obesity together affect glucose tolerance. Burghen et al. showed a positive correlation between HA and hyperinsulinemia [20]. This association has an important place in clinical diagnosis. Paradisi et al. [21] showed a positive correlation between IR and endothelial dysfunction. However, Mather et al. [22] showed normal endothelial function in young obese women with IR. In our study, a strong correlation was found between HOMA-IR, total testosterone, and fasting blood glucose levels in patients with PCOS. However, unlike other studies, no correlation was found between DHEAS and SHBG levels. Likewise, in IR and CIMT there was no difference. This may be because the average age group in our study was younger, and the BMI was lower than other studies, and therefore there was no correlation between IR and CIMT. IR is also associated with the FG score, and it is thought to be related to simultaneously high levels of free and total testosterone.

Serum homocysteine levels, a cardiovascular risk factor, also showed a positive correlation with CIMT in some studies. Furthermore, IR, impaired glucose tolerance, obesity, type 2 diabetes, and dyslipidemia were found to be more common in patients with PCOS, showing that this group of people is an indicator of inflammation [23]. In our study, a significant difference in serum homocysteine levels between PCOS patients and controls was observed. However, the correlation was not significant.

Another notable feature of PCOS is that it is accompanied by dyslipidemia. Studies have shown a positive correlation between dyslipidemia and cardiovascular diseases [24,25]. In our study, no significant correlation was found between dyslipidemia and CIMT when evaluating in the PCOS group. This is thought to be because the patient population we investigated was a young group and did not have any additional risk factors.

## Conclusions

In conclusion, according to the results of this study, a relationship between increased CIMT, dyslipidemia, elevated levels of homocysteine, and PCOS can be seen. Since these are risk factors for atherosclerosis and cardiovascular disease and CIMT is an indicator, PCOS patients should not be evaluated and managed only from a gynecological perspective. They need a multidisciplinary approach and management of their condition in order to avoid long-term adverse cardiovascular effects.
